# Impact of Post-Translational Modifications of Crop Proteins under Abiotic Stress

**DOI:** 10.3390/proteomes4040042

**Published:** 2016-12-21

**Authors:** Akiko Hashiguchi, Setsuko Komatsu

**Affiliations:** 1 Faculty of Medicine, University of Tsukuba, Tsukuba 305-8577, Japan; hashiguchi.akiko.ge@un.tsukuba.ac.jp; 2 National Institute of Crop Science, NARO, Tsukuba 305-8518, Japan

**Keywords:** post-translational modifications, crop, abiotic stress, soybean

## Abstract

The efficiency of stress-induced adaptive responses of plants depends on intricate coordination of multiple signal transduction pathways that act coordinately or, in some cases, antagonistically. Protein post-translational modifications (PTMs) can regulate protein activity and localization as well as protein–protein interactions in numerous cellular processes, thus leading to elaborate regulation of plant responses to various external stimuli. Understanding responses of crop plants under field conditions is crucial to design novel stress-tolerant cultivars that maintain robust homeostasis even under extreme conditions. In this review, proteomic studies of PTMs in crops are summarized. Although the research on the roles of crop PTMs in regulating stress response mechanisms is still in its early stage, several novel insights have been retrieved so far. This review covers techniques for detection of PTMs in plants, representative PTMs in plants under abiotic stress, and how PTMs control functions of representative proteins. In addition, because PTMs under abiotic stresses are well described in soybeans under submergence, recent findings in PTMs of soybean proteins under flooding stress are introduced. This review provides information on advances in PTM study in relation to plant adaptations to abiotic stresses, underlining the importance of PTM study to ensure adequate agricultural production in the future.

## 1. Introduction

Living organisms are frequently faced with abiotic stress conditions such as low nutrient availability, salinity, extreme temperatures, drought, and high ultraviolet irradiation [1]. Organisms must adjust their physiology and development to assure their survival under changing conditions within a time range in diurnal, seasonal, annual periodicity or even on the order of several years to several tens of years [1,2]. As sessile organisms, plants have to endure a wide variety of environmental stresses while staying in the same habitat during their entire life cycle. To achieve this, plants have evolved many intricate mechanisms that involve hormonal actions of gibberellin, abscisic acid (ABA), ethylene, and jasmonate [3]. Hormonal cross-talk modulates through interaction among signaling components determine plant responses to abiotic stresses when they occur simultaneously [4]. Modulation of protein activity occurs at the level of transcription which determines protein abundance, followed by regulation at levels of translation, and post-translation. Among them, post-translational modifications (PTMs) are crucial because they can bring about fine-tuning of protein function, localization, half-life, and interactions to mitigate the potential damage of environmental stresses [5,6,7].

Human population growth and global industrialization are factors that affect food crop production through altering atmospheric conditions, changing traditional systems of crop production, and modifying environmental soils using chemical fertilizer while increasing the demand for stable crop supply [8]. To solve food security issues by stabilizing crop production, technology development for increased productivity is required in unstable environments. Research is being conducted to understand response characteristics of useful plants by obtaining data on global change in plant physiology [9]. Based on omics data represented by proteomic profiles, attempts for mathematical modeling of plant response mechanisms against stimulation were underway with a focus on the prediction of plant response under field conditions [10,11,12]. However, studies for crops have lagged behind those of the model species because of genetic diversity of important crops and resulting scarcity of genomic information or knowledge on response mechanisms in each species [13,14]. In this review, research on protein PTMs in crops is introduced, though it is still an emerging field. Plant proteomic technologies for analysis of representative PTM and involvement of PTMs are described in plant acclimation to environmental change such as phosphorylation, glycosylation, acetylation, and succinylation. Proteins for which the role of their PTMs are extensively studied will be described as well. Finally, recent advancement in PTM study in crops under abiotic stress is referenced, taking soybean seedlings under flooding stress as an example.

## 2. Techniques for Detection of Post-Translational Modifications in Crops

### 2.1. Difference Gel Electrophoresis and Gel-Free Approaches

Difference gel electrophoresis (DIGE) is a protein labelling and separation technique for quantification in mixtures of two or more samples using optical fluorescence of differentially labelled proteins. 2D DIGE combined with mass spectrometry analysis of each spot has been traditionally used to quantify changes in crop protein abundance in response to environmental changes because of its relatively high sensitivity [15]. Crop response to the circadian system was analyzed in rice seedlings by the technique to identify light-regulated oscillating proteins [16]. For studying protein PTMs, the ability of DIGE to separate one particular protein into several modified isoforms is an advantage. In order to acquire meaningful biological interpretation using gel-based protein separation approaches, in-depth multivariate analysis is required using protein spots correlation on the gels, and, therefore, the data processing method should be optimized [17]. There is an attempt to improve the bioinformatics software so that biologists can easily identify correlations between gel spot patterns and physiological states [18]. Newly developed software is expected to accelerate defining a panel of biomarkers of certain physiological states and understanding the molecular mechanism of plant responses to environmental stress. Regarding the improvement of experimental practices, the assessment of protein comigration that impairs the accuracy of protein quantification led to modified protein separation techniques. The addition of a third step of separation was introduced in DIGE recently (3D DIGE) [19]. Using 3D DIGE, proteins that undergo SUMOylation during leaf development were newly identified, expanding understanding of endogenous plant SUMOylome [19]. Development of image analysis software and improvement of protein separation techniques increase accurate identification and reliability of comparative analysis of different physiological states.

Development of MS-based technology along with completion of genome sequencing has made liquid chromatography-mass spectrometry (LC-MS) a routine technique in the field of plant biology as well as crop proteomics [20]. For quantitative proteomics by a gel-free MS-based method, sample preparation is critical to the success of downstream analytical methods. In order to quantify changes in the relative amount of proteins that reflect the magnitude of the intracellular signal, label-based protein quantification is available [21]. It includes both in vivo labeling and in vitro labeling. Stable isotope labeling by amino acids in cell culture (SILAC) is one of the in vivo labeling techniques that is applied to plants, and it makes use of metabolic reactions of sample organisms [22,23]. In vitro labeling includes techniques such as an isotope-coded affinity tag (ICAT) and an isobaric tag for relative and absolute quantitation (iTRAQ) [24,25]. The acquisition of mass signals in large-scale proteomic studies employs data-dependent acquisition (DDA) strategies where a fixed number of intact peptides (MS^1^) are selected as precursors and subsequently analyzed as fragments by tandem mass (MS/MS) [21]. On the other hand, data-dependent acquisition (DIA) does not require obtaining MS^1^ data for selection but uses all precursors, so it became a sensitive alternative to DDA [26]. Improvement of multiplexed quantification by the DIA method is underway to make the technique compatible with label-based protein quantification [27]. In crops challenged by abiotic stress, a label-free method is widely used that compares spectra of common peptides between samples [20]. In soybeans under flooding stress, a gel-free label-free method was applied and revealed the role of energy management and protein homeostasis for survival under submergence [28,29,30]. Employment of a rapidly advancing proteomic approach to crop science will help identify players for stress tolerance.

### 2.2. Lectin Blot

Lectins are a class of carbohydrate-binding proteins. Many cell surface proteins are glycoproteins with various sugar chain length, 3D structure, and composition of sugar residues [31]. Glycoproteome differs from each other depending on the physiological status, and accordingly glycosylation analysis has become an important issue in proteomic research [32]. For the analysis of glycoproteins that characterize the particular physiological status of the cells, glycan-specific lectins are used. Lectins that have different specificities to glycans are a useful tool for the selection of proteins with specific glycosylation. They enable detection of a global change of glycosylation pattern of proteins by polyacrylamide gel electrophoresis (PAGE) combined with Western blotting that uses appropriate fluorescently-labeled lectins [31]. Frequently used lectins are Concanavalin A from *Canavalia ensiformis* and Wheat Germ Agglutinin, which recognize mannose type *N*-glycans and *N*-acetyl glucosamine that are further decorated by sialic acid, respectively [31]. In addition to the lectin blot, lectins are used for glycoprotein fractionation and purification by lectin affinity chromatography [33]. However, further analysis of glycoproteins by mass spectrometry technique requires liberation of glycans from the core protein. To avoid this step, a novel technique called lectin microarray was established for easy differential glycan profiling in 2005 [34]. In lectin microarray, multiple lectins that are immobilized on a glass interact with proteins bearing different glycans to produce specific spot patterns. This technique was applied for detecting probes for human pluripotent stem cells in the biomedical field [35]. Utilization of this technique in crops will expand our knowledge on glycoscience of crop development and physiology.

### 2.3. Enrichment of Post-Translational Modifications

PTM occurs only in a set of proteins within the entire proteome. Therefore, enrichment of modified proteins is required to overcome the difficulties of the detection of low abundance proteins. Several techniques were developed and applied for detecting modified proteins that are otherwise lost in the crowd of abundant proteins. Enrichment technologies make use of immunoaffinity, protein charge, or hydrophobicity [36,37]. For analysis of phosphoproteins, the anti-phosphotyrosine (Y) antibodies can be immobilized on a solid support to allow the enrichment of modified proteins from complex protein mixtures. Methods that use positively charged metal ions or metal oxides are techniques known as immobilized metal affinity chromatography (IMAC) or metal oxide affinity chromatography (MOAC), respectively. Combined usage of commercially available IMAC or MOAC with interaction chromatography methods can improve recovery late of modified peptides of interest in the animal research field [37]. A study relating to crop production was performed by Li et al. using polymer-based metal ion affinity capture using germinating rice seeds [36]. The study identified nuclear transcription factors that relay developmental signals to trigger the activation of a cascade of sequential tissue differentiation [36]. Recent advances in methods to separate phosphopeptides enabled analysis of proteins that undergo multiple protein phosphorylations. It is possible to analyze proteins separately according to the number of phosphorylations by sequential elution from IMAC (SIMAC), a method combing two chromatographic steps with different metal species [38]. Various PTMs other than phosphorylation were studied by immuno-enrichment and affinity chromatography: acetylation [39], glycosylation [40], and ubiquitination [41]. For unstable PTMs like nitrosylation, labeling by S-biotinylation is applied followed by separation with affinity columns as seen in the biotin switch assay [42]. In addition, selective labeling and enrichment of nitrosylated proteins were developed using an isotope-coded cysteine thiol-reactive multiplex reagent, cysTMT^6^, in place of biotin [43]. Advancement in protein enrichment techniques will accelerate deeper analysis of PTMs.

## 3. Systems for Post-Translational Modifications in Crops under Abiotic Stress

### 3.1. Phosphorylation

Reversible protein phosphorylation is a PTM that functions as a crucial regulatory mechanism in many biological processes [44]. Phosphorylation is mostly found in hydroxylated amino acids such as serine (S), threonine (T) and Y residues. Studies of target proteins of S/T and Y phosphorylation in crop plants include wheat, maize, and sugar beet under salt stress [45,46,47] and wheat and several beans under drought stress [48,49]. These environmental stresses mediated by protein phosphorylation can influence crop productivity by retarded growth or imparted fertility. A phosphoproteomic analysis in maize identified hundreds of male sterility-associated proteins which were assumed to form protein complexes with kinases [50]. Regarding the regulation of phosphorylation, the protein kinase gene family as well as the protein phosphatase gene family have been widely studied in model plants and agronomically important crops such as wheat and maize [51,52,53]. It was revealed that plants do not possess typical protein tyrosine kinase genes [51]. However, protein tyrosine phosphatases were discovered in crops like tomato [54]. Although Y phosphorylated proteins exist in relatively low abundance, which is 4.2% in *Arabidopsis*, 2.9% in rice, and 1.3% in *Medicago*, Y phosphorylation plays an important role in plant responses to a changing environment [55]. Ectopic induction of drought stress-inducible plant and fungi atypical dual-specificity phosphatase (PFA-DSP1) can change stress response pattern against drought of tobacco plants [56]. In chestnut trees under cold acclimation, DSP4 shows upregulated expression, suggesting a phosphorylation-mediated mechanism to withstand cold stress [57]. An increasing number of large-scale phosphoproteomic datasets is now available for in-depth pathway analyses [58,59]. A study of barley CPK2, a kinase that works in calcium-mediated drought response pathways, indicated that modulation of phosphorylation by CPK2 overexpression leads to drought sensitivity [60]. Soybean BZL2 is a target protein of phosphorylation, and disruption of a phosphorylation site of this protein resulted in enhanced brassinosteroid signaling [61]. Identification of phosphorylation targets of stress-responsive kinases such as CPK2 and reconstruction of affected pathways may facilitate our understanding of how stress tolerance is achieved in challenged plants.

### 3.2. Glycosylation

Glycosylation is one of the most abundant PTMs required for plants, with a wide repertoire of biological implications. The main types of this PTM are the *N*- and *O*-glycosylations that occur at Asp (D) residue or either at S/T or Y residues, respectively [62]. Cell-surface proteins are mostly glycoproteins. Proteins for secretory pathway are modified by an *N*-glycan through the process of *N*-glycosylation in the endoplasmic reticulum (ER) and follow a pathway which promotes efficient folding with the assistance of calnexin (CNX), calreticulin (CRT), and heat shock proteins [62]. In plants, environmental stresses such as osmotic/drought stress as well as ER stress commonly activate cell death signal across species [63]. However, overexpression of heat shock proteins that facilitates the folding process in ER as molecular chaperones can rescue cellular damage and increase tolerance against salt stress in tomato [64]. These results suggested that the detrimental outcome occurring under various environmental stresses is in part due to defects in protein folding for which aberrant *N*-glycosylation is responsible. In crop species, rice challenged by abiotic-stresses like oxygen deprivation forms aerenchyma in the cortical area of roots by shifting cell wall integrity and inducing programed cell death [65]. The β1,2-xylosyltransferase is an enzyme that catalyzes the transfer of xylose to the *N*-glycans in Golgi apparatus whose defect disrupts root aerenchyma formation. The rice mutant with defective xylosyltransferase is susceptible to low heat or osmotic stresses [66]. The α1,3-fucosyltransferase catalyzes the transfer of fucose to the *N*-glycan cores. Loss of function of fucosyltransferase resulted in reduced gravitropic responses in rice [67]. The above-mentioned results indicate that tolerance against environmental stresses in rice depends on how accurately proteins are transported and localized using *N*-glycans as delivery tags. The addition of glycan moieties to proteins is strictly regulated to form heterogeneous sugar chains with different branching patterns. Step by step editing of sugar chains consist of the attachment and trimming of sugar residues, and this process plays a role in seed development of *Raphanus sativus* [68]. Descriptions of site-specificity and added sugar chains are underway with the help of novel bioinformatics tools for glycan identification, fragmentation spectra annotation, and quantitation, although an example for this was shown in palm trees but not yet in other crops [69]. Extensive research on the influence of sugar chain structure on protein function needs to be undertaken to develop a complete picture of the plant adaptation to the environment.

### 3.3. Acetylation

Protein acetylation is a well-known post-translational regulatory mechanism that regulates gene expression. The acetylation status of histone and non-histone proteins is regulated by several factors like physiological stresses, reactive oxygen species, and infectious diseases. Protein acetylation occurs in two distinct forms; one is non-reversible Nα-terminal modification by Nt-acetyltransferases, and the other is reversible ε-amino group modification of lysine (K) by K acetyltransferases (KATs) and K deacetylases (KDACs) [39]. Histones are one of the target proteins of KATs and KDACs. Modifications on histones will be described later in this review. Studies of crop acetylome were performed in rice, wheat, and soybeans [39,70,71]. Nallamilli et al. described global K acetylation in rice [39]. It was shown that a wide range of cellular processes were affected by K acetylation from metabolic processes, signal transduction, RNA processing, protein translation, and stability. Proteins related to cell death were included among 44 proteins identified as acetylated proteins in rice [39]. This was followed by a study in wheat, another important cereal crop [70]. In total, 277 wheat proteins were identified, having a large proportion of them involved in energy production [70]. Biochemical pathways for energy production are dynamic processes under stress conditions. Protective metabolic adaptations include altered photosynthesis and glycolysis to adjust energy balance and let the plant survive [72]. As described above, protein acetylation controls the metabolic adaptations via modifying non-histone metabolic enzymes. Furthermore, it participates in the shift of energy production via changing gene expression. A rice histone deacetylase, OsSRT1, was shown to bind to specific genomic loci to promote expression of genes related to metabolic processes [73]. Further analysis dealing with correlation of stress conditions, physiological status of the cells, and acetylomes can link environmental stress cues to the regulation of metabolic pathways.

### 3.4. Succinylation

Succinylation is a PTM where a succinyl group is added to a K residue of a protein molecule. It is a relatively newly identified PTM, and it was first identified through the analysis of a mass shift in *Escherichia coli* isocitrate dehydrogenase followed by identification of diverse proteins that undergo this modification [74]. Protein succinylation is dynamic under diverse cellular conditions and is the most crucial protein PTM involved in the regulation of plant growth and development [75]. During germination, rice seed undergoes dynamic rearrangements of metabolism and succinylation status if its proteome changes [75]. It was shown that, in rice seedlings, succinylated proteins are enriched in functional categories such as citrate cycle, glycolysis/gluconeogenesis, ribosome, and carbon fixation with notably high coverage in pathways involved in the conversion of glucose to pyruvate [76]. The seedling leaves of *Brachypodium distachyon*, a grass family plant also exhibited the PTM in carbon metabolism pathways [76]. According to the comparison of succinylated proteins across species by Zhen et al., a high degree of conservation of succinylation was suggested among eukaryotes including humans, *E. coli*, and plants [75]. Extensive characterization of the tomato succinylome identified that several sites of histone H3 (K14, K56, K79, and K122) are targets for succinylation [77]. Because these are sites that were also modified in other organisms including humans, mice, *Drosophila melanogaster*, and *Saccharomyces cerevisiae*, evolutionarily conserved protein succinylation is assumed to be a central regulatory mechanism of biological processes [76]. Elucidating elaborate regulation of important metabolic pathways by PTMs is crucial for plant adaptation to stressors and enhancement of functionality of crops.

### 3.5. Other PTMs

Other well-studied PTMs in crop abiotic stress systems include oxidative modifications such as carbonylation, *S*-nitrosylation, and Tyr-nitration [78]. These PTMs have a broad range of target proteins, but most prominent targets are related to energy production as shown in *Vigna mungo* under aluminium stress [79]. Salt stress leads to *S*-nitrosylation on respiratory, photorespiratory, and some oxidative stress-related enzymes in pea plants [42]. Cold stress also brings about *S*-nitrosylation in *Brassica juncea* [80]. As carbon monoxide radicals or nitric oxide radicals are produced in response to environmental stress as signaling molecules, elucidation of the link between stress-responsive signaling molecules, proteins that undergo PTMs, and plant responses generated by the PTMs is to be desired.

## 4. Post-Translational Modifications of Crop-Proteins

### 4.1. Histone

The packaging of DNA with histone into nucleosomes and chromatin in higher order structures allows tight regulation of gene expression [81]. The ability of plants to cope with abiotic environmental stresses relies on flexible mechanisms for re-programming a set of gene expression under different stressors [81]. Nucleosomal dynamics is regulated by histone variants with PTMs such as acetylation [82], methylation [82], phosphorylation [83], ubiquitination [84], and succinylation [81]. Description of histone PTMs is accumulating in *Brassica napus* and useful plants such as tomatoes [85,86,87]. Acetylation and methylation are well studied among them as important PTMs that alter epigenetic status of plants. Acetylation of histones is promoted in maize by gibberellin and suppressed by ABA, two phytohormones involved in response to abiotic stresses [88]. In salt-stressed maize, plant response is accompanied by an increase in the global acetylation levels of histone H3 at K9, which was shown to control increased cell wall proteins like expansins and the plasma membrane proton pump [89]. There is evidence in *Arabidopsis* that epigenetic variations can pass on to subsequent generations as seen in heat stress response [90]. In addition, this mechanism is utilized to remember the mode of gene expression against previous encounters with environmental stresses, thus ensuring rapid adaptation in subsequent stresses as shown in maize [91]. Analysis of PTMs of histone and gene expression that is controlled by each histone modification will advance our understanding of long-term plant response to external stimuli.

### 4.2. Tubulin

The plant cortical microtubule array has been postulated to be of importance for plant cell morphogenesis that depends on directional cell expansion. It is a dynamic structure in which individual tubulin subunits are constantly polymerizing and dissociating [92]. Tubulin α was shown to be increased in African rice cultivars that are exposed to salt stress as well as in Asian rice cultivars under heat stress [93,94]. A single copy of a tubulin protein bears multiple modification sites and can be modified by multiple types of PTMs including phosphorylation [95], acetylation [96,97], and polyglutamylation [97]. The PTM pattern is tissue-specific as indicated in developing maize [97]. It is stress-responsive as revealed in soybeans under cadmium stress [98]. Among PTMs that are observed in tubulins, phosphorylation is a modification which rapidly conveys stress signal to change plant physiological status. It was shown that the phosphorylation response of α tubulin occurs within a few minutes after hyperosmotic stress treatment and promotes depolymerization of microtubules in rice [95]. A tubulin complex-related serine/threonine protein kinase named OsNek6 was regulated by ubiquitin-dependent proteolysis in response to drought stress [99]. These results obtained from crops suggest an elaborate mechanism that controls tubulin and microtubule function, although they are limited in number. Multiple PTM events that are closely intertwined with each other are assumed to play important roles. To obtain deeper insight into mechanisms of tubulin control by PTMs, research using *Arabidopsis* is mentioned below. A kinase named propyzamide-hypersensitive 1 is a phosphatase which was recently shown to possess a novel atypical kinase domain [100]. Phosphorylation of α-tubulin by propyzamide-hypersensitive 1 at T349 inhibits tubulin recruitment to microtubules within ten minutes after exposure to osmotic stress in *Arabidopsis*. Interestingly, the tubulin kinase activity is normally suppressed by the intrinsic phosphatase activity of propyzamide-hypersensitive 1 [100], suggesting that propyzamide-hypersensitive 1 can act as a switch for microtubule reorganization when a plant experiences environmental stresses, although the clue for this switch for enzymatic activity is not yet elucidated. Plant hormones such gibberellic acid are known to modulate microtubule reorientation and tubulin isotypes [101]. Enzymes like propyzamide-hypersensitive 1 with dual functions may pivot abiotic stress-mediated signaling pathways from static status to acute response to optimize plant physiology in order to ensure survival, using tubulins as mediators. Further studies on PTMs of tubulin and its upstream enzymes in crops will add new insights into the mechanism of stress perception and response determination.

## 5. Post-Translational Modifications of Soybean Proteins under Flooding Stress

### 5.1. Phosphorylation in Nucleus

Soybeans are a valuable legume crop and are rich in protein and oil. However, they are susceptible to water deficiency and submergence [72]. Quantitative proteomic studies on soybean seedlings under flooding stress revealed that the plants cope with the stress by anoxia-responding proteomic adaptation [102,103]. Perception of the external stimuli is generated via calcium signaling [104]. In soybeans under flooding stress, phosphoproteins have a crucial role in stress signal transduction. Treatment of soybeans with abscisic acid can improve flooding tolerance [105], and mediators of ABA signaling are identified as nuclear phosphoproteins as mediators of early responses to the stress including zinc finger/broad-complex, tramtrack and bric a brac (BTB) domain-containing protein 47, glycine-rich protein, and rRNA processing protein Rrp5 [105]. These results underline the importance of that phosphorylation and nuclear translocation of the intracellular mediator of ABA signaling in early response to flooding stress. Yin et al. [5] also identified 146 proteins in total as factors affected in common between ABA-treated and flooding-tolerant mutant soybeans, revealing biological processes including protein synthesis and RNA regulation playing a central role in tolerance induction in initial flooding stress. Further analysis is required to identify how ABA-induced translocation of phosphorylated proteins brings about changes that help to establish flooding tolerance.

### 5.2. Glycosylation in Endoplasmic Reticulum

Mustafa et al. [106] revealed that flooding stress affects the glycosylation level of several proteins in germinating soybeans. Disturbance of the secretory pathway was implicated by the decrease in glycoproteins that were classified in secretory pathways and degradation. On the other hand, cell wall proteins were accumulated. Calcium signaling and cell wall metabolism have been implicated in flooding tolerance in soybeans for many years [104,107]. Improved cell wall integrity was observed during survival and recovery from flooding [105,108]. Cell wall proteins were glycosylated *N*-glycoproteins and *O*-glycoproteins, whose highly heterogeneous sugar chains play important roles in cell–cell recognition during development or content-dependent intercellular signaling [109]. The glycosylated cell wall proteins are modified within the ER. As disruption of glycosylation results in perturbations in protein deposition and ER stress, ER proteomics was performed in soybean root tips under submergence [30]. In the study, protein glycosylation level was lowered along with changes in enzymes for glycosylation. Factors for protein folding in ER, such as CNX and protein disulfide isomerase-like proteins, markedly decreased, suggesting that submergence affects soybean seedlings by disrupted protein folding of glycosylated protein through CNX/CRT cycle. In stressed plants, proteins are endocytosed and targeted to the trans-Golgi network and then recycled back to the plasma membrane, which is an essential process for environmental responses [110]. Whether a disturbance in protein glycosylation affects the protein recycling process is an interesting question that can help to elucidate soybean response to flooding stress.

### 5.3. Ubiquitination in Cytosol

Maintenance of protein homeostasis, or proteostasis, is now well recognized to be related to stresses that trigger disease development in animals and plants [111]. Removal of misfolded or unnecessary proteins by ubiquitin/proteasome systems is essential for averting proteotoxicity or unintended cellular response. Ubiquitin/proteasome-mediated degradation involves Ub-activating enzyme (E1), Ub-conjugating enzyme (E2 or UBC), and ubiquitin ligase (E3) [6]. In soybean seedlings under submergence, the apical meristem of root tips is a highly vulnerable organ [72]. Soybean upregulates one of the two paralogs of ubiquitin ligase E3 in flooded roots [102] as well as two subunits of COP9 signalosome, CSN4, and CSN5, for proteolysis of ubiquitin-conjugated proteins [6]. Although COP9 signalosome activation is independent from oxygen deprivation, active protein degradation seems an important regulatory mechanism of soybean root response to survive flooding.

A coordinated reaction in gene expression increases survival chances by plant tolerance. The phytohormone ethylene and jasmonate act antagonistically to regulate plant acclimation against stresses including drought, salt, ozone toxicity, cold, and flooding, by modulating transcriptional responses [112,113,114]. Several nuclear transcription proteins were identified so far that function downstream of ethylene in soybeans under flooding [115]. A large number of instances were reported where ubiquitin/proteasome-mediated degradation was involved in regulating phytohormone signaling [116]. Interference of ethylene and jasmonates signaling by *SUBMERGENCE1A*, an ethylene-response-factor-like gene, improves rice flooding tolerance and delays leaf senescence in rice [117]. It was supposed that activation of ubiquitin/proteasome-mediated degradation targets components of phytohormone signaling to elicit appropriate gene expression in flooded soybeans.

## 6. Conclusions

The research on crop protein PTMs and their role in regulating stress responses are just beginning to become a subject of active study. Advances in affinity chromatography make it possible to increase the percentage of modified proteins and, together with separation technique, contribute to data accumulation of proteins that undergo PTMs under abiotic stresses in both model plants and crops. The molecular mechanism for which protein PTMs affect the total outcome of plant response has partly been made clear in model plants by identifying interacting partners or analyzing gene expression that is controlled downstream of modified proteins [100,118]. Significant progress has been made in histone PTMs regarding how these marks are being read and contribute to forming “memory” of the repeated stresses [90,91]. Overview of systems governed by PTMs as stress-responsive factors is available for phosphorylation, glycosylation, acetylation and ubiquitination in *Arabidopsis* and monocots like rice. However, the information in other PTMs in crop species, especially dicot crops, are still limited and descriptive. Therefore, the ultimate function of many of these PTMs requires further intensive study. PTMs in soybean plants subjected to flooding stress were introduced as a pioneering study of analysis of dicot crops. In conclusion, this review is a brief summary of crop PTMs and their associated functions. Revolutionary developments in laboratory techniques and system biology would encourage future research and rapid deciphering of novel functions of crop protein PTMs in plant adaptation to environmental changes.
